# Augmentative Biocontrol in Natural Marine Habitats: Persistence, Spread and Non-Target Effects of the Sea Urchin *Evechinus chloroticus*

**DOI:** 10.1371/journal.pone.0080365

**Published:** 2013-11-15

**Authors:** Javier Atalah, Grant A. Hopkins, Barrie M. Forrest

**Affiliations:** Cawthron Institute, Nelson, New Zealand; Institute of Marine Research, Norway

## Abstract

Augmentative biocontrol aims to control established pest populations through enhancement of their indigenous enemies. To our knowledge, this approach has not been applied at an operational scale in natural marine habitats, in part because of the perceived risk of adverse non-target effects on native ecosystems. In this paper, we focus on the persistence, spread and non-target effects of the sea urchin *Evechinus chloroticus* when used as biocontrol agent to eradicate an invasive kelp from Fiordland, New Zealand. Rocky reef macrobenthic assemblages were monitored over 17 months in areas where the indigenous algal canopy was either removed or left intact prior to the translocation of a large number of urchins (>50 ind.·m^−2^). Urchin densities in treated areas significantly declined ∼9 months after transplant, and began spreading to adjacent sites. At the end of the 17-month study, densities had declined to ∼5 ind.·m^−2^. Compared to controls, treatment sites showed persistent shifts from kelp forest to urchin barrens, which were accompanied by significant reductions in taxa richness. Although these non-target effects were pronounced, they were considered to be localised and reversible, and arguably outweigh the irreversible and more profound ecological impacts associated with the establishment of an invasive species in a region of high conservation value. Augmentative biocontrol, used in conjunction with traditional control methods, represents a promising tool for the integrated management of marine pests.

## Introduction

Biological invasions can have profound impacts on ecosystem functioning, by altering community structure, native species richness and ecological processes [1], [2]. The magnitude and extent of these impacts vary across temporal and spatial scales [3], but they can be extensive and irreversible [4]. Historically, a range of tools has been used to mitigate impacts associated with marine pests, including physical [5], [6], chemical [7], [8] and biological [9], [10] treatments. Compared to terrestrial and freshwater systems, control or eradication tools used in the marine environment are often simplistic, labour intensive and implementation at a large-scale is generally not feasible [6]. In this context, there is growing interest in developing integrated, cost-effective and environmentally-sound marine pest management tools. Biological control (biocontrol), specifically augmentative biocontrol with indigenous agents, stands out as a promising approach, but is one that has not yet been used in natural marine habitats at an operational scale [9], [11]. Augmentative biocontrol can be considered in the context of two recognised strategies [12], [13]. The first is the inoculation approach, in which biocontrol agents are released with the expectation that they will multiply and reduce pest populations for an extended period [12], [14]. The other is an inundation strategy which relies exclusively on shorter term effects of the release of a large number of agents [12].

Critical to the success of augmentative biocontrol are considerations of the ecology, population dynamics and behaviour of specific control agents before their release in natural habitats [15], [16]. This includes assessment of organisms in terms of their persistence, and spread to new habitats or areas [17]. Simultaneously, it is necessary to consider their direct or indirect non-target effects; the release of indigenous natural enemies into novel environments may lead to collateral impacts such as reduced biodiversity and altered ecosystem functioning [18].

Urchins have long been recognised as a strong structuring force of benthic communities throughout coastal areas worldwide [19], [20], [21]. Previously, urchin species have been trialled as biocontrol agents only in small-scale experiments both on artificial structures [22], [23] and in natural habitats [24]. The New Zealand sea urchin, *Evechinus chloroticus* (hereafter *Evechinus*), has been used during trials on suspended artificial structures, and has been found to control a wide range of algae and invertebrate species, including several high profile marine pests [25].

More recently, *Evechinus* was used as part of a multi-agency response in a New Zealand fiord to eradicate an incursion of the non-indigenous Asian kelp *Undari*a *pinnatifida* [26], which provided a unique opportunity to evaluate urchin biocontrol at an operational scale. An overview of the eradication programme and its outcomes will be described elsewhere by the agencies involved. In this paper, we describe the persistence and spread of *Evechinus* that were transplanted using an inundation approach during the eradication effort, and quantify the associated changes in subtidal benthic assemblages at transplant sites. Such knowledge provides information on both the non-target effects of *Evechinus* as a biocontrol agent, and also its potential efficacy as a control agent for other species. Finally, we consider some of the benefits and limitations of using urchins for managing marine pests in natural habitats.

## Methods

### Ethic statement

All required permits for this study were obtained from the Department of Conservation and Ministry for Primary Industries. No locations were privately-owned and the studies did not involve endangered or protected species.

### Study sites

**Figure 1:**
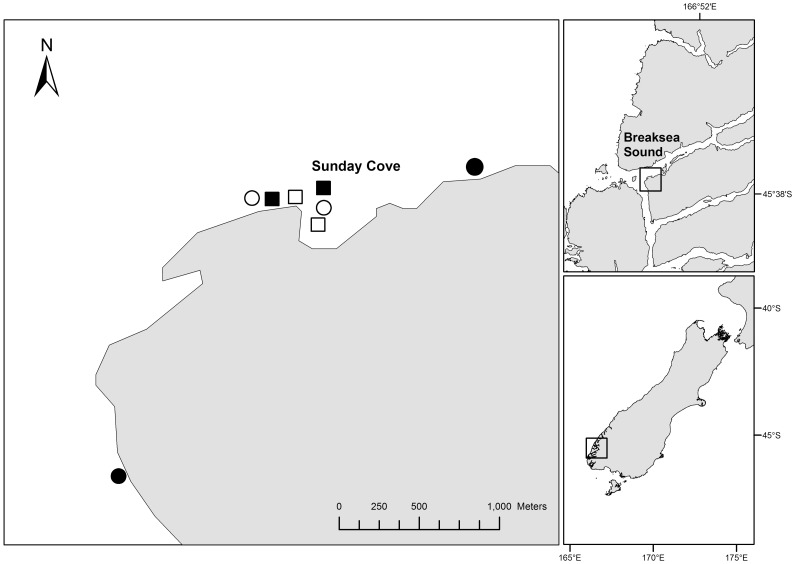
Map of the study sites. Filled circles denote ‘Control’, empty circles ‘Adjacent’, empty squares ‘No Canopy’ and filled squares ‘Canopy’ sites

The study was conducted in Sunday Cove (45°36′S, 166°44′E), a small, shallow embayment of approximately 2.1 ha surface area in Breaksea Sound, Fiordland (Fig. 1). Fiordland is subjected to high rainfall (5300–6300 mm yr^−1^) that creates a permanent low-salinity surface layer (<30 ‰) of variable depth in the fiords, but is typically 1–2 m deep in outer fiord areas such as Sunday Cove [27]. This low-salinity layer results in stratified habitats, with the surface layer dominated by euryhaline organisms. Deeper areas are characterised by dense ‘kelp forest’ habitats dominated by large canopy forming brown macroalgae, such as *Ecklonia radiata* and *Carpophyllum flexuosum* (hereafter *Ecklonia* and *Carpophyllum*, respectively), and diverse understory assemblages [28], [29]. At mid-depths sea urchins may remove large areas of kelp forest and form habitats termed ‘urchin barrens’, dominated by encrusting algal forms.

During the eradication programme, a total of 30,000–35,000 *Evechinus* individuals (mean test diameter  = 11.3 cm, ±1.5 SE) were collected [26] by divers from sites in the outer reaches of Breaksea Sound. They were transported by boat in ∼70 L self-draining plastic containers within ∼1 h to four treatment sites: two ‘Canopy’ and two ‘No Canopy’ sites. These sites were in established kelp forest habitats at depths of 5–7 m within Sunday Cove (Fig. 1). The plastic containers were then submerged by divers and *Evechinus* were manually placed within each of the treatment sites. Sampling sites were then established in a circular formation at each of the treatment sites, with sampling occurring within a 5 m radius of a weighted central marker. At two of the sites (No Canopy sites), the algal canopy was removed before adding *Evechinus*, while at the other two sites (Canopy sites) the algal canopy was left intact. The treatment sites (i.e. both Canopy and No Canopy) were chosen based on previous locations of mature *Undaria pinnatifida* individuals found during the eradication programme [26]. Two ‘Adjacent’ sites within Sunday Cove (<50 m from the treatment sites) and two ‘Control’ sites outside of Sunday Cove (>500 m from the treatment sites) were also selected (Fig. 1). Adjacent sites were established to assess the effects on areas contiguous to the treatment sites, as a result of the anticipated urchin spread. Sites were chosen so that they were geographically interspersed and, when possible, chosen randomly from a larger pool of candidate sites. *Evechinus* were transplanted to the No Canopy sites before the first sampling occasion, resulting in a mean density of 52.5 ind.·m^−2^ (±2.7 SE). Whereas, at the two Canopy sites, *Evechinus* was added in August 2011 (after the first sampling occasion), resulting in a mean density of 51.9 ind.·m^−2^ (±2.8). Natural mean densities of *Evechinus* at the Adjacent and Control sites were 0.1 (±0.1) and 0.3 (±0.2) ind.·m^−2^, respectively.

### Sampling

Sites were sampled on four occasions: in August 2011 immediately before *Evechinus* were transplanted to the two Canopy sites, then again in October 2011, April 2012 and December 2012. Counts of stipes of *Ecklonia* and *Carpophyllum*, as well as *Evechinus* individuals were obtained from ten random 1 m^2^ quadrats at each of the eight sites. To sample the understory community, eight haphazardly placed 0.24 m^2^ photo–quadrat images were taken with a digital underwater camera. Before taking each photograph, blades of large brown algae and individual *Evechinus* were moved outside each quadrat so that the understory was visible. At the two Canopy sites, the spread of *Evechinus* from the initial seeding location was recorded by measuring their densities within 1 m^2^ quadrats (n = 5) at three locations: the site perimeter (edge) and two distances beyond (5 and 10 m).

The photo-quadrat images were analysed using the Coral Point Count software [30], with 50 stratified random points overlaid on each image. All sessile taxa >1 mm in size were identified to the lowest possible taxonomic level and their percentage cover estimated. Taxa that could not be identified at species or genus level were grouped by morphological criteria (Table 1).

**Table 1:** List of taxa (not including *Evechinus*) recorded on the photo quadrats at the study sites. Column ‘Group’ refers to variable names used in Fig. 5.

Taxon	Category	Group
**Echnidermata**		
*Patiriella regularis*	Echinoderm	Asteroidea
*Australostichopus mollis*	Echinoderm	Holothuroidea
Holothurian unid.	Echinoderm	Holothuroidea
**Brachiopoda**		
*Terebratella sanguinea*	*Terebratella sanguinea*	Brachiopoda
**Porifera**		
*Cymbastela tricalyciformis*	Sponge	Demospongiae
*Crella incrustans*	Sponge	Demospongiae
*Dysidea* sp.	Sponge	Demospongiae
*Haliclona* sp.	Sponge	Demospongiae
*Latrunculia fiordensis*	Sponge	Demospongiae
Encrusting sponge unid.	Sponge	Demospongiae
Erect sponge unid.	Sponge	Demospongiae
*Strongylacidon conulosum*	Sponge	Demospongiae
*Tethya* sp.	Sponge	Demospongiae
**Cnidaria**		
Hydroid unid.	Hydroid	Hydrozoa
*Anthothoe albocincta*	Anthozoan	Anthozoa
*Corynactis australis*	Anthozoan	Anthozoa
**Chlorophyta**		
*Caulerpa brownii*	Green algae	Bryopsidales
*Codium gracile*	Green algae	Bryopsidales
Green filamentous	Green algae	Ulvaceae
*Ulva* spp.	Green algae	Ulvaceae
**Phaeophycea**		
*Carpophyllum flexuosum*	Brown canopy	Fucales
*Ecklonia radiata*	Brown canopy	Laminariales
Brown filamentous algae	Brown filamentous	Phaeophyceae
*Carpomitra costata*	Brown herbaceous	Sporochnales
*Dictyota* spp.	Brown herbaceous	Dictyotales
*Halopteris* spp.	Brown herbaceous	Leptothecata
*Zonaria turneriana*	Brown herbaceous	Littorinimorpha
*Microzonia velutina*	Brown herbaceous	Cutleriales
*Ralfsia verrucosa*	Encrusting algae	Ralfsiales
**Rhodophyta**		
Coralline crustose algae	Coralline crustose algae	Corallinales
Coralline turfing algae	Coralline turfing algae	Corallinales
*Hildenbrandia* spp.	Encrusting algae	Hildenbrandiales
Red complex foliose algae	Red complex foliose algae	Rhodophyta
Red filamentous algae	Red filamentous algae	Rhodophyta
Red laminar foliose algae	Red laminar foliose algae	Rhodophyta
**Bryozoa**		
*Beania* sp.	Bryozoan	Bryozoa
Erect bryozoan	Bryozoan	Bryozoa
Catenicellid bryozoan	Bryozoan	Bryozoa
*Margaretta barbata*	Bryozoan	Bryozoa
*Membranipora membranacea*	Bryozoan	Bryozoa
**Tunicata**		
*Cnemidocarpa* bicornuta	*Cnemidocarpa bicornuta*	Tunicata
*Aplidium* sp.	Colonial ascidian	Tunicata
*Botrylloides* sp.	Colonial ascidian	Tunicata
*Botryllus* sp.	Colonial ascidian	Tunicata
Colonial ascidian	Colonial ascidian	Tunicata
Didemnidea	Colonial ascidian	Tunicata
*Diplosoma* spp.	Colonial ascidian	Tunicata
**Mollusca**		
*Cellana radians*	Gastropod	Mollusca
Gastropod unid.	Gastropod	Mollusca
*Mytilus galloprovincialis*	Bivalve	Mollusca
**Polychaeta**		
*Galeolaria hystrix*	Polychaeta	Polychaeta
*Spirobis* spp.	Polychaeta	Polychaeta
**Other**		
Bare rock	Inorganic	Other
Sand	Inorganic	Other
Shell hash	Inorganic	Other
Biofilm	Organic	Other
Detritus	Organic	Other
Other organic	Organic	Other

### Data analyses

To investigate treatment effects, data were analysed with Population-Averaged Generalized Estimating Equations (PA-GEEs), an extension of Generalized Linear Models for correlated data [31]. This procedure was used because sites were sampled repeatedly through time and, although randomly positioned quadrats were sampled at each time, data could not be treated as independent in conventional analyses. PA-GEEs provide estimates of model parameters and standard errors that take into account correlated observations to avoid spurious correlations. Additionally, PA-GEEs estimate a scale parameter, which is an adjustment for overdispresion. A first order autoregressive AR(1) working correlation matrix was used in all of the analyses, assuming observations from a given site separated by one sampling occasion were likely to be more similar than those separated by longer time periods. Models were fitted for data on densities of *Evechinus*, *Ecklonia* and *Carpophyllum* using a Poisson error distribution and a log-link, whereas Gaussian errors were used for the number of taxa and Shannon diversity. The number of taxa and Shannon's diversity index were calculated from the data obtained in the photo-quadrats, having first removed organic (i.e. detritus and other organic) and inorganic (i.e. sand, bare rock and shell hash) cover data (Table 1). The experimental design comprised three factors: ‘Treatment’ (fixed, four levels: Canopy, No Canopy, Adjacent, Control), ‘Time’ (fixed, four sampling dates: August 2011, October 2011, April 2012 and December 2012) and ‘Site’ (random, two levels, nested in factor Treatment). Comparisons were based on the Control treatment level and the first sampling occasion (i.e. August 2011) as reference points. For the spread experiment data from the two Canopy sites, the effect of time and distance on *Evechinus* density was also examined by fitting a PA-GEE model as described above. Time (months since addition) and distance (meters from the edge of each site) were included as covariates in the model, while site was included as a categorical factor. All PA-GEE models were selected using the Quasi Likelihood under the Independence Model criterion (QIC), a modification of the Akaike's Information Criterion for PA-GEEs models [32], and validated by inspecting the deviance residuals. PA- GEEs models were run using the library *geepack* [33] in the software R [34].

Differences in assemblage structure between experimental treatments were tested using PERMANOVA [35] based on Bray–Curtis similarities of log(*x*+1) transformed data. The same experimental design was used as described above for the univariate analyses, with factors ‘Treatment’ (fixed, four levels), ‘Time’ (fixed, four levels) and ‘Site’ (random, two levels, nested in factor Treatment). Significant terms were then investigated using *a posteriori* pair-wise comparisons with the PERMANOVA *t* statistic and 999 permutations. SIMPER analysis [36] was used to identify the percentage contribution of each species (or taxon) to the observed differences between assemblages in the different treatments. Taxa that consistently discriminated between treatments (ratio of the average dissimilarity and standard deviation ≥3) were displayed as vectors in principal coordinate ordination plots (PCO).

## Results

### Changes in *Evechinus* densities

**Figure 2:**
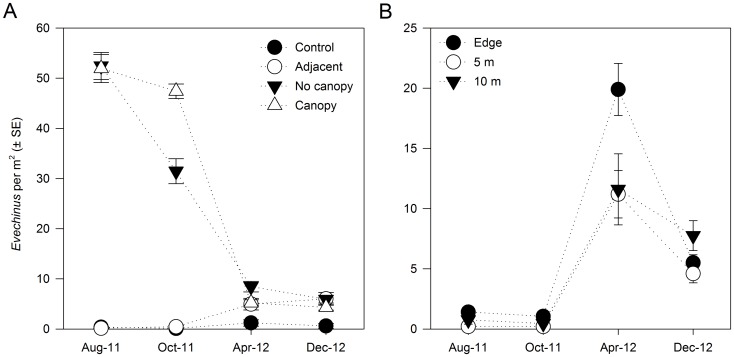
Changes in sea urchin density through time for each A) treatment and B) distance. Data were pooled across sites (n = 20).

Field observations indicated that *Evechinus* individuals quickly relocated to suitable areas after being transplanted, moving from sandy, biogenic and vertical (or steeply sloping) habitats into relatively flat rocky areas. Over time, significant reductions in *Evechinus* densities were observed at both Canopy and No Canopy sites in relation to the initial mean densities of 51.9 (±2.8 SE) and 52.5 (±2.7) ind.·m^−2^, respectively (Fig. 2A, Table 2). At these sites, densities rapidly declined between October 2011 and April 2012, but in December 2012 had stabilised at mean values of approximately 5 ind.·m^−2^. *Evechinus* densities at the Adjacent sites increased from mean values of 0.15 (±0.1) and 0.45 (±0.3) ind.·m^−2^ in August and October 2011, to 4.95 (±1.16) and 6.0 (±1.26) ind.·m^−2^ in April and December 2012, respectively (Fig. 2A, Table 2). Urchin densities at the Control sites remained stable throughout the study at <5 ind.·m^−2^ (Fig. 2A). Overall, there was no significant spatial variation among sites within each treatment (Table 2).

**Table 2:** Results of Population-Averaged Generalized Estimating Equations (PA-GEEs) examining the effects of time and treatment on the density of *Evechinus*, *Ecklonia* and *Carpophyllum*.

	*Evechinus*			*Ecklonia*			*Carpophyllum*			Number of taxa			Shannon diversity		
	Estimate	SE	*P*	Estimate	SE	*P*	Estimate	SE	*P*	Estimate	SE	*P*	Estimate	SE	*P*
Site	0.11	0.07		0.62	0.19	**	−0.04	0.13		1.07	0.50	*	0.05	0.09	
Oct-11 x Adjacent	2.27	1.10		−0.10	0.31		0.25	0.22		2.54	1.22	*	0.52	0.18	**
Apr-12 x Adjacent	2.13	1.17	*	−0.86	0.60		−0.21	0.75		0.44	1.58		0.36	0.16	*
Dec-12 x Adjacent	2.91	0.94	**	−1.81	0.52	***	−1.98	0.77	*	−0.11	2.02		0.47	0.39	
Oct-11 x Canopy	1.16	0.92		−45.89	0.82	***	−44.51	0.84	***	−5.63	1.59	***	−0.66	0.19	***
Apr-12 x Canopy	−3.64	0.94	***	−45.64	0.88	***	−44.28	0.84	***	−6.09	1.66	***	−1.00	0.20	***
Dec-12 x Canopy	−3.21	0.95	***	−45.64	0.83	***	−43.81	0.86	***	−3.91	2.27	*	−0.10	0.39	
Oct-11 x No Canopy	0.73	0.94		−0.04	1.00		−0.35	1.02		2.84	1.93		0.25	0.46	
Apr-12 x No Canopy	−3.17	0.93	***	0.21	1.05		−0.12	1.02		3.13	2.27		0.72	0.44	
Dec-12 x No Canopy	−2.91	0.94	**	0.22	1.01		0.35	1.04		3.12	2.52		0.80	0.56	
Scale Parameter	3.09	0.48		0.91	0.17		0.96	0.26		6.00	0.72		0.17	0.02	
Correlation Parameter	0.20	0.14		0.43	0.12		0.07	0.07		0.44	0.06		0.55	0.05	

Main effects for Treatment and Date are not shown, as all interactions were significant. Treatment effect comparisons are in reference to the controls and the first sampling occasion in August 2011. *P<0.05, **P<0.01 and ***P<0.001.

Reductions in *Evechinus* densities at the two Canopy transplant sites were reflected in the spread experiment results. The patterns of spread evident from density changes were similar between the two Canopy sites, and more strongly related to time since *Evechinus* transplant (*P*<0.001) than distance from the sites (Fig. 2B, Table 3). In August and October 2011, densities at all distances from the Canopy sites were <1.5 ind.·m^−2^. However, concomitant with the reduced densities at Canopy sites in April 2012 (see Fig. 2A), there was an outward movement of *Evechinus* from the point of initial transplant, reflected by increased densities at the spread sites (e.g. ∼20 ind.·m^−2^ at the 10 m site) (Fig. 2A). By December 2012, densities had decreased to mean values between 4.6 (±0.7) and 7.7 (±1.2) ind.·m^−2^ for all distances (Fig. 2B), which were comparable to the residual densities at the Canopy sites (Fig. 2A).

**Table 3:** Population-Averaged Generalized Estimating Equations (PA-GEEs) results examining the effects of time, distance and site on *Evechinus* density. Log-link and Poisson errors. **P<0.01.

	Estimate	SE	*P*
Distance	−0.08	0.07	
Time	0.07	0.01	**
Site	−0.28	0.16	
Distance x Time	0	0.01	
Scale Parameter	11.7	1.57	
Correlation Parameter	0.64	0.05	

### Effects of *Evechinus* on canopy forming algae

**Figure 3:**
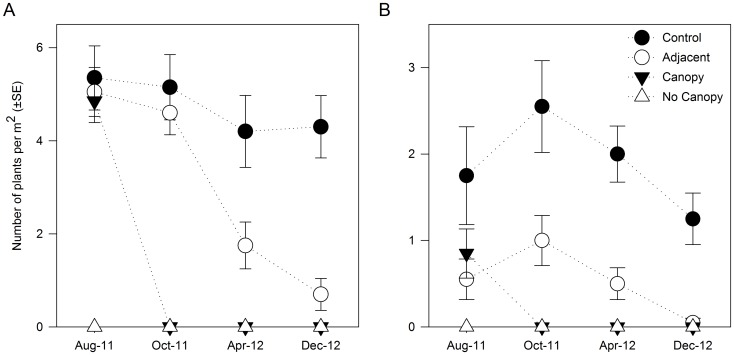
Treatment effect through time on density of A) *Ecklonia radiata* and B) *Carpophyllum flexuosum*. Data were pooled across sites (n = 20).

Mean densities of *Ecklonia* at the beginning of the experiment (August 2011) were approximately 5 ind.·m^−2^ for all treatments, with the exception of the No Canopy sites where the canopy had been manually removed by divers prior to *Evechinus* transplant (Fig. 3A). After *Evechinus* transplant to the Canopy sites (i.e. August 2011), there were significant reductions in *Ecklonia* densities at the Canopy and Adjacent sites over time (Fig. 3A, Table 2). Canopy sites were completely denuded of *Ecklonia* by the first resampling (i.e. October 2011). However, at the Adjacent sites there was a gradual decline in *Ecklonia* over time to 0.7 ind.·m^−2^ in December 2012 (Fig. 3A), simultaneous with the gradual increase in *Evechinus* densities at those sites (Fig. 2A). Additionally, there was a significant variation in densities of *Ecklonia* between sites within given treatments (Table 2).

The spatio-temporal pattern of density change for *Carpophyllum* was comparable to that described for *Ecklonia*. Mean densities of *Carpophyllum* at the beginning of the study ranged from 0.5 to 1.8 ind.·m^−2^ for all treatments except the No Canopy sites where the algae were manually removed (Fig. 2B). Densities at the Control sites remained relatively stable throughout the study, whereas no individuals were recorded at the Canopy sites from October 2011 onwards (Fig. 3B, Table 2). At the Adjacent sites there was a small increase in *Carpophyllum* densities in October 2011, after which densities gradually declined to zero by December 2012.

### Changes in the number of taxa, diversity and structure of understory assemblages

**Figure 4:**
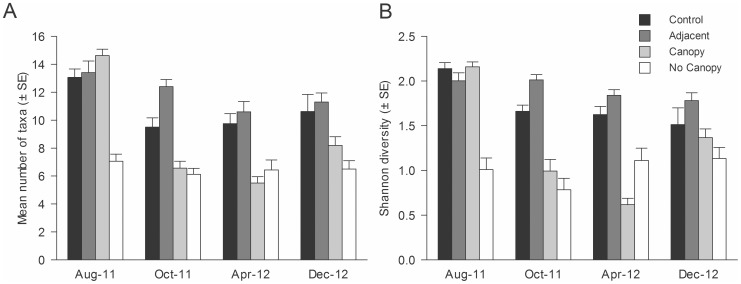
Treatment effect through time on A) mean number of taxa and B) Shannon diversity. Data were pooled across sites (n = 16).

Significant changes in the mean number of taxa through time were observed in response to the urchin treatments, with Canopy and No Canopy sites having approximately half the number of taxa recorded in the Control and Adjacent sites from October 2011 onwards (Fig. 4A, Table 2). At the start of the experiment in August 2011, the mean number of taxa at the Control, Adjacent and Canopy sites was 11.9, 12.0 and 13.1 (±0.6, 0.8 and 0.5, respectively). In contrast, at the No Canopy sites where *Evechinus* had already been transplanted, the mean number of taxa was 4.6 taxa (±0.4). In October 2011, the mean number of taxa at the Canopy sites was significantly reduced to 4.0 (±0.5), while no significant changes were observed in the other treatments (Table 2). Patterns observed both in April and December 2012 were similar to that described for October 2012, with significantly fewer taxa at both Canopy and No Canopy sites compared to the Control and Adjacent sites (Fig. 4A, Table 2). In December 2012, both of the urchin-treated sites displayed a slight increase in their mean number of taxa compared to April 2012, but this trend was not significant. This pattern was confirmed by a similar trend observed for the Shannon's diversity index (Fig. 4B, Table 2); both Canopy and No Canopy sites had lower diversity in October 2011 and April 2012 compared to the controls, which then increased in December 2012 (Fig. 4B).

**Figure 5:**
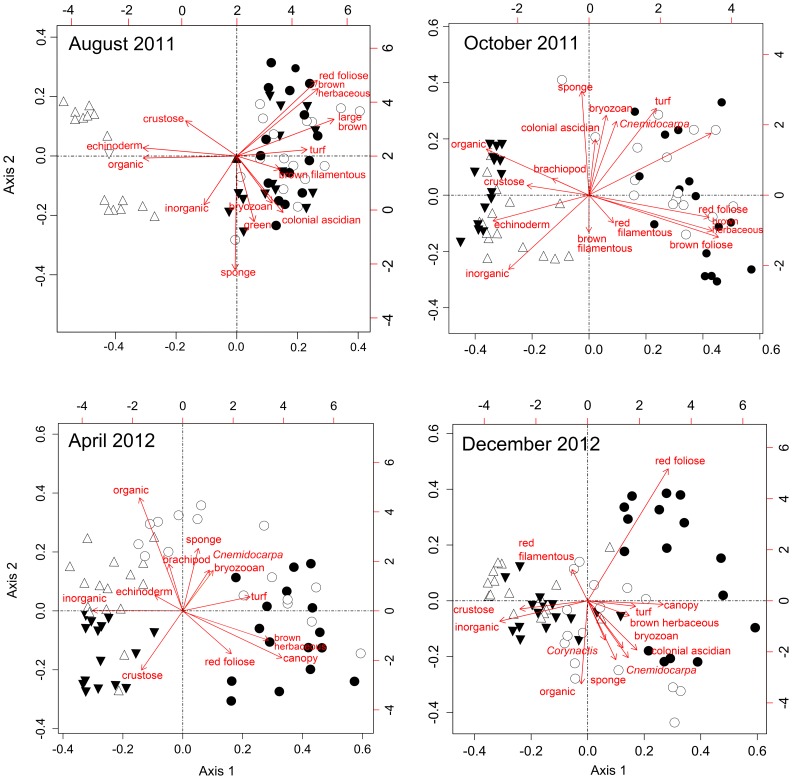
Principal coordinates ordination (PCO) biplots illustrating treatment effects on the understory macrobenthic assemblages. Plots are based on Bray-Curtis similarities of log(*x*+1) transformed data

Significant shifts in understory community structure were observed in response to the treatments over time, as indicated by a significant Treatment x Time interaction (*P*<0.05, Table 4), despite significant spatial variability at the site-scale within treatments (*P*<0.001, Table 4). The PCO plots illustrate the overall pattern among treatments for each of the sampling dates (Fig. 5). At the beginning of the study in August 2011, the understory assemblages at the Control, Adjacent and Canopy sites (before *Evechinus* addition) did not differ significantly (pair-wise comparison *P*>0.05). Assemblages were characterised by a mixture of large canopy forming brown algae, turfing coralline algae, red foliose algae, small brown herbaceous algae (both filamentous and foliose forms), green algae, colonial ascidians and bryozoans. These taxa collectively accounted for >75% of observed similarities among these sites. In contrast, the No Canopy sites were significantly different to all other treatments (pair-wise comparison *P*<0.05). These sites resembled an urchin barren, being largely dominated by crustose algae, echinoderms (other than *Evechinus*; see Table 1), organic and inorganic matter and the solitary ascidian *Cnemidocarpa bicornuta*.

**Table 4:** PERMANOVA of differences in benthic assemblage structure based on Bray-Curtis dissimilarities of log transformed data (using 9999 permutations).

Source	df	MS	F	*P*
Treatment	3	52999	6.92	**
Time	3	18060	6.25	***
Site (Treatment)	4	7653.5	14.75	***
Treatment x Time	9	7885.2	2.73	***
Time x Site (Treatment)	12	2888.3	5.56	***
Residual	224	519.02		

During the second sampling occasion (October 2012) both Canopy and No Canopy sites exhibited urchin barren characteristics. These sites were dominated by crustose algae, echinoderms (e.g. sea stars), the brachiopod *Terebratella sanguinea* and both organic and inorganic matter, which together accounted for >90% of their similarity (Fig. 5). In contrast, Adjacent and Control sites remained characterised by kelp forest assemblages and were significantly different to the treated sites (pair-wise comparison *P*<0.05). A large proportion of the similarity of the Adjacent and Control sites was accounted for by red foliose algae, large brown fucoid algae, crustose algae, small brown filamentous and foliose algae, coralline turf, and suspension feeding invertebrates such as sponges and bryozoans.

By the third sampling occasion (April 2012), the Control sites remained significantly distinct from the urchin treated sites (pair-wise comparison *P*<0.05), but assemblages at the Adjacent sites (where *Evechinus* densities had increased) were transitional between the control and treated sites. The substratum cover at Adjacent sites had similarities with an urchin barren, and was characterised by bare rock, organic detritus, crustose algae, coralline turf, *Cnemidocarpa bicornuta* and encrusting sponges (Fig. 5). Nonetheless, the Adjacent sites still had a diverse complement of species that was comparable to the controls, highlighting that the patterns in Fig. 5 reflect trends in taxon structure rather than composition. Assemblages in the Canopy and No Canopy sites continued to be largely dominated by crustose algae, inorganic and organic matter and echinoderms, such as the sea cucumber *Australostichopus mollis* and the seastar *Patiriella regularis*. A similar pattern was observed during the last sampling occasion (December 2012), with only the Control sites differing to the treated sites (Fig. 5, pair-wise comparison *P*<0.05), but assemblages at the Adjacent sites were marginally distinct to the treated sites (pair-wise comparison *P*<0.1). The Control sites remained dominated by kelp forest assemblages, while the Canopy and No Canopy sites were characterised by urchin barren assemblages (crustose algae, organic and inorganic matter and *Cnemidocarpa bicornuta*). However, a relatively larger cover of red and green ephemeral algae was recorded in these sites compared to previous sampling occasions.

## Discussion

### Changes in *Evechinus* densities

Over the course of the study *Evechinus* densities declined by one order of magnitude at the treated sites, from ∼50 to 5 ind.·m^−2^. Adjacent sites had similar urchin densities to the treated sites after nine months. Natural densities of *Evechinus* are variable, although in urchin barrens these are typically around 5 ind.·m^−2^ [37], [38], [39]. Nonetheless, densities of over 50 ind.·m^−2^ have been documented in natural systems [40], which is comparable to the enhanced transplant densities in the present study. *Evechinus* can adapt to situations of high conspecific densities and associated low food availability, through adjustment in growth and reproductive output [41]. As such, they can persist at high densities (e.g. 5 ind.·m^−2^) for many years and be replenished by recruitment of new individuals. Recruitment of new *Evechinus* was not observed during the course of this study (authors' personal observation). Additionally, increased recruitment in response to locally enhanced adult densities would not be expected, as recruitment dynamics in this species are largely influenced by larval advection and other abiotic factors that operate at the whole-fiord scale [42].

The temporal reduction in densities within the treated sites is consistent with the spread experiment, which confirmed that *Evechinus* moved >10 m away from the edge of the transplant areas in a relatively short period of time (<9 months). Based on tagged individuals, Dix [40] concluded that *Evechinus* individuals dispersed over maximum distances of 5 m in six months, and hypothesised that their movement was related to food availability. The relatively higher spread observed in the present study is possibly related to the scarcity of food at the treated sites, where very high *Evechinus* densities quickly created barren areas. Biocontrol agents with low spread rates may necessitate the redistribution of individuals or a high density of release for effective control to be exerted. On the other hand, high rates of spread may lead to a reduction in densities in target areas to a point where control is ineffective. Given such issues, it has been argued that the use of species with intermediate rates of spread is likely to maximize the probability of successful augmentative biocontrol [17].

### Effects of *Evechinus* on benthic assemblages

At the treated sites, *Evechinus* rapidly (in <3 months) grazed the canopy forming algae (*Ecklonia* and *Carpophyllum*) and most of the understory assemblages, including turfing, foliose and filamentous algae, bryozoans, colonial ascidians and sponges. *Evechinus* preferentially graze on laminarian algae, specifically *Ecklonia* [43], but can consume a wide range of other algal and invertebrate species at relatively high rates [37], [43].

The low-richness urchin barren assemblage that resisted *Evechinus* grazing pressure included encrusting algal forms, and to some extent the brachiopod *Terebratella sanguinea* and the solitary ascidian *Cnemidocarpa bicornuta*. These findings are consistent with the well-recognised role of *Evechinus* as a primary driver of community structure of shallow subtidal rocky reef assemblages in New Zealand [37], [39], [41], [43], [44]. Elsewhere, increased abundances of *Evechinus* are strongly related to reductions in canopy forming algae, understory algae and encrusting invertebrates [37]. In this respect, it is of interest that *Evechinus* densities at Adjacent sites were similar to treated sites at the end of the study (almost certainly a reflection of spread from the treated sites), yet the range of taxa remained comparable to controls. This contrast likely reflects the different site histories; despite Adjacent and treated sites reaching comparable *Evechinus* densities, the Adjacent sites never experienced the intense grazing pressure that would have resulted from the initial high density urchin transplant at treated sites.

### Broader perspective on benthic effects

The dramatic changes to benthic assemblage structure highlights the potential for substantial non-target effects associated with generalist biocontrol agents. This issue is particularly relevant in areas of high conservation value such as in the present study. Some trepidation around the use of biocontrol has resulted from early experiences, particularly classical biocontrol in terrestrial systems, where procedures were less regulated and non-target effects were less considered [16]. It is common for marine pest management programmes (e.g. physical or chemical treatments) to be implemented with a lack of proven and cost-effective control tools, on the basis that prompt response will give the greatest chance of success [5], [6], [45]. In such instances, ecological risks are likely to be relatively unimportant; for example, perhaps at worst leading to local-scale effects that are reversible. By contrast, the use of marine biocontrol agents that have not been exhaustively evaluated in terms of their efficacy and non-target effects is probably not advisable.

Crucial to the assessment of non-target effects is the magnitude and spatio-temporal scale of the impacts. The choice of *Evechinus* as a biocontrol agent was based largely on previous ecological knowledge of the species (e.g. high consumption rates, generalist feeding habits and wide distribution). Relevant to the issue of non-target effects in the present study is that *Evechinus* were transplanted from urchin barrens within the same fiord as the transplant sites. In this sense, the regional population remained largely unchanged; it was simply redistributed. Although the non-target effects of transplant were dramatic, and occurred outside of the initial transplant sites (e.g. at Adjacent sites), they could be reversed by the removal or destruction of *Evechinus* from treated areas. However, based on *Evechinus* longevity (10–15 years) and growth rates (1–2 cm yr^−1^) [46], it can be expected that the transplanted population will likely decline over the subsequent years. Additionally, the continued spread of *Evechinus* will likely reduce densities to levels that enable large brown algae and understory assemblages to re-establish in the longer term. Existing studies suggest that the threshold density for recovery is <2 ind.·m^−2^ [47]. Based on these considerations, it is expected that the biocontrol effects of *Evechinus* will be localised and reversible. In natural systems, shifts from urchin barren to kelp forest have also been related to the recovery of sea urchin predators [48], mass sea urchin mortality due to storms [49] and diseases [50], no-take marine reserves protection [39], [44] and harvesting [47].

As well as the spatial and temporal scales of non-target effects, distinction also needs to be made in terms of the species that are affected by biocontrol [18]. Impacts on species should not be considered equally; special consideration should be given to endangered species, species that provide crucial ecosystem services as well as species of commercial value [51]. The taxonomic resolution described in the present study does not enable such issues to be fully addressed. At a broad level, the assemblages found at the study sites were comparable to those inhabiting similar habitats (i.e. kelp forest) in the wider area [28], [29], [52]. However, it is clear that ecologically important species were adversely affected as a result of the *Evechinus* transplants. For example, *Ecklonia* is an ecosystem engineer that provides habitat, nurseries and food (both directly and through detrital additions to the food chain) to many other species [53], [54]. However, in the context of marine pest management, the significance of localised non-target effects from biocontrol must be weighed up against the potentially irreversible and regional-scale effects resulting from the establishment of an invasive species.

### Broader consideration of the utility of urchins in biocontrol

Despite the potential for non-target effects, the present study illustrates the potential efficacy of augmentative biocontrol. Such approaches, when used in conjunction with more traditional management measures [55] may provide a useful way forward for future invasive species responses. For example, diver-based pest response programmes would benefit from the use of generalist biocontrol agents that denude canopy, by aiding visual searches. This benefit may prove crucial to the success of marine pest eradication attempts, as one of the primary problems with manual diver removal is the failure to detect individuals before they successfully reproduce [56], [57].

It is conceivable that a similar biocontrol approach could be applied in other regions, using the same or different urchin species, or in fact other generalist grazers or predators. The use of such taxa is not only restricted to the control of algae, but can clearly be extended to a range of other pests, both invertebrates and algae. For example, *Evechinus* efficiently removed colonial ascidians; although this was a non-target effect in the present context, it is relevant in a wider context that ascidians are an invertebrate group that can show invasive traits [58].

For *Evechinus* specifically, applications are restricted to mainly hard bottom substrata, because the species has limited grazing activity in soft bottom and biogenic habitats [40]. These habitats may constitute a refuge for the settlement of invasive algae. Similarly, high substrate rugosity or biogenic complexity may create important refuges, where invasive species may escape urchin grazing pressure. This situation was evident in the present study, and may have contributed to persistence of some species that would otherwise have been vulnerable. In the context of the study region itself, vertical walls were common in the shallowest zones of the treated sites, and the wider area was subject to variable or reduced salinity in surface layers that likely created conditions that urchins could not tolerate [59].

More broadly, augmentative biocontrol approaches are likely to have greatest efficacy and practicality in local-scale applications that target spatially restricted areas. It would not be practical to use this strategy for pests that were geographically dispersed, certainly because of logistical constraints, and perhaps because of unacceptable large-scale non-target effects. Additionally, augmentative biocontrol is perhaps more suited to the purpose of population control rather than eradication, except where used alongside a broader suite of tools [60]. A final consideration is that, because the settlement and dispersal of invasive species can be facilitated by disturbance [61], urchin barrens have been suggested as favourable habitats for being colonised by marine pests [62]. This could result in a counteractive effect in which the use of biocontrol enhanced, rather than controlled, the spread of a target pest. For example, in Tasmania the invasive alga *Undaria pinnatifida* can successfully recruit and persist in urchin barren areas [63]. In this context, it is crucial that monitoring is continued in treated areas after the cessation of an eradication programme, or the potential removal of the control agents is considered.
